# Mental health issues, risks and barriers to care for Bangladeshi children and adolescents: insights from lived experiences

**DOI:** 10.1093/inthealth/ihaf091

**Published:** 2025-08-25

**Authors:** Md Omar Faruk, Kamal Uddin Ahmed Chowdhury, Sabiha Jahan, Erminia Colucci

**Affiliations:** Department of Clinical Psychology, University of Dhaka, 5017 Arts Building, Dhaka 1000, Bangladesh; Department of Psychology, Louisiana State University, 236 Audubon Hall, Baton Rouge, LA 70803, USA; Department of Clinical Psychology, University of Dhaka, 5017 Arts Building, Dhaka 1000, Bangladesh; Department of Clinical Psychology, University of Dhaka, 5017 Arts Building, Dhaka 1000, Bangladesh; Department of Psychology, Faculty of Science and Technology, Middlesex University London, London, UK

**Keywords:** adolescents and children, Bangladesh, barriers, mental health issues, qualitative exploration, risks

## Abstract

**Background:**

Mental health issues among Bangladeshi children and adolescents are increasing due to sociocultural factors. Despite its importance, in-depth research is scarce. This study addresses the gap by exploring mental health issues, risk factors and barriers to accessing care in rural and urban Bangladesh through qualitative methods.

**Methods:**

We conducted semi-structured interviews with 38 participants across both rural and urban areas, including 12 in-depth interviews with children and adolescents, 10 key informant interviews with caregivers and 2 focus group discussions with community representatives, educators and healthcare providers. All interviews were transcribed verbatim and a thematic analysis was employed to identify key themes. In addition to the interviews, we utilized observational data and yearly records from ADD International Bangladesh, a community-based mental health organization, to provide contextual depth and triangulate findings.

**Results:**

The findings revealed three themes: mental health issues such as anxiety, depression, suicidal ideation and substance abuse; risk factors including device overuse, family conflicts and substance availability; and barriers to care such as lack of services, low mental health literacy and stigma from religious and societal beliefs.

**Conclusions:**

The study provides an in-depth understanding of mental health issues, risk factors and barriers to care in rural and urban areas of Bangladesh. The findings enhance the understanding of the mental health status of young people and can inform policy development and the formation of suitable psychosocial interventions.

## Introduction

Mental health issues are prevalent in people irrespective of age, gender, socio-economic status, race, ethnicity or geographic location. According to the World Health Organization (WHO), approximately 1 in 8 people globally (970 million) are living with a mental health disorder.^1^ Statistics further suggest that by 2021, 13.9% of the global population was affected by mental disorders, contributing to 17.2% of the total years lived with disability worldwide.^2^ After analysing information from 205 countries and territories, the Global Burden of Disease 2019 data showed that globally mental disorders continued to be one of the leading causes of burden, with no evidence of a decrease since 1990.^3^ The coronavirus 2019 (COVID-19) pandemic has increased the prevalence rates of mental disorders, particularly depression and anxiety.^4^ Recent data also suggest that globally women are more likely than men to be affected by mental illnesses (e.g. depression: 5.5–6% and 3–4% in women and men, respectively) and anxiety (4.7% and 2.8% in women and men, respectively).^5^ Children and young people are no exception to developing mental health issues. For example, recent data show globally 10–20% of children and adolescents experience mental disorders, with a 2019 estimate of 1 in 7 (14%) adolescents ages 10–19 y being affected (166 million total: 89 million boys, 77 million girls).^6^ Mental disorder prevalence increased with age, ranging from 6.80% in children ages 5–9 y to 13.96% in those ages 15–19 y.^7^ The prevalence rates are disproportionately higher for children and adolescents living in low- and middle-income countries (LMICs), with recent estimates suggesting prevalence ranges from 12 to 29%.^6^ A meta-analysis conducted in 2022 showed that 19.8% of children/adolescents in LIMCs had at least one mental disorder.^8^ It is important to note that suicide is the third leading cause of death among youth ages 15–19 years worldwide and the leading cause in some regions such as Southeast Asia.^9^ These conditions among children and adolescents have exponentially increased globally during and after the pandemic.^8,10,11^

Mental health issues in children and adolescents include externalizing (e.g. aggression and oppositional defiance) as well as internalizing (e.g. anxiety and depression), behaviours that persist through childhood and adolescence into adulthood.^12–14^ For example, researchers showed that children who experienced adversities during their formative years are more likely to have an increased risk of developing anxiety, mood and substance use disorders in adulthood.^15^ The researchers also added that these problems may continue to have an impact on adults even when children are taken out of the problematic environments. Yet the mental health issues of children and adolescents are largely understudied and often ignored in many LMICs, where the development of mental health services is disadvantaged by a lack of government policy, inadequate funding and a shortage of trained and skilled mental health professionals.^16^ This is particularly relevant for Bangladesh amid the growing presence of mental health issues in children and adolescents.^17^

In Bangladesh, the National Mental Health Survey conducted in 2019 reported that 14% of children ages 7–17 y suffer from mental health problems and approximately 95% do not seek psychiatric consultation.^18^ Another study found the prevalence rates of moderate to severe levels of depression and anxiety among school-going adolescents were 26.5% and 18.1%, respectively.^19^ Risk factors for depression include unsatisfactory sleep patterns, grade level, sex, cigarette smoking (especially between ages 15–16 y) and perceived lack of good relationships with friends were risk factors for anxiety among adolescents.^19,20^ Another recent study investigating the prevalence and associated factors of depression among adolescent boys and girls found the overall prevalence of moderate, moderately severe and severe depression was 5.4%, 1.1% and 0.1%, respectively.^21^ Poverty (e.g. lack of food and consumption of unfortified oil and non-iodized salt), excessive television viewing time and insufficient recreation (e.g. physical exercise) were associated with depression. Moderate to severe depressive and anxiety symptoms among school adolescents in urban, semi-urban and rural areas in Bangladesh were found to be 30.1% and 20%, with females reporting these symptoms more than their male counterparts.^22–24^ In both cases, socio-economic status, geographic location, inadequate physical activity and a substantial amount of time spent using mobile devices were found to be significantly associated with anxiety among adolescents.

The prevailing view of mental health issues further worsens the already significant prevalence of these problems among children and adolescents. The prevailing perception toward mental health issues primarily revolves around widespread stigma and discrimination resulting in a wide treatment gap.^25,26^ Many people in Bangladesh suffer in silence and experience isolation and discrimination.^26^ For instance, the National Mental Health Survey demonstrated that people with mental health problems reported that visiting mental health professionals might result in labelling with derogatory terms such as mad.^18^ As a result, people, including children and adolescents, with mental health issues use a range of traditional healthcare providers such as faith healers (pirs and fakirs), homeopathic practitioners and retail medicine sellers as their first point of contact for healthcare.^27^ Refraining from seeking appropriate mental healthcare further increases prevalence rates and treatment gaps. While the mental health issues and associated risk factors among children and adolescents identified in the studies mentioned above have primarily emerged from quantitative research, there is limited research on in-depth exploration of these issues, risk factors and prevailing attitudes in rural and urban regions of Bangladesh. This study aims to fill that gap.

## Methods

We followed the standards for reporting qualitative research.^28^ We conducted in-depth interviews (IDIs) with children and adolescents and key informant interviews (KIIs) with caregivers. We also conducted focus group discussions (FGDs) with community leaders, educators and local government representatives.

### Study context

The study was conducted in four rural coastal areas (Rampal, Gotapara, Bastali and Shatgambuz union) of Bagerhat, a southwestern region in Bangladesh. Data were also collected from urban areas of Dhaka (Badda and Bauniabadh), the capital city of Bangladesh, and Jashore Sadar, also a southwestern region. The present study was conducted as part of a longitudinal community-based mental healthcare project called ‘Bridging the Gaps: Strengthening Mental Health Support for Child and Young People’, led by ADD International Bangladesh, a non-governmental developmental organization working for people with and without disabilities toward ensuring healthcare and education in rural and urban areas. Three organizations (i.e. Innovation for Wellbeing Foundation [IWF], Disabled Children’s Foundation [DCF] and Nasiruallah Psychotherapy Unit) collaborated with ADD International Bangladesh. IWF and DCF has field offices in the areas where the study was conducted.

### Sampling strategy

We employed a purposive sampling strategy. Purposive sampling enables researchers to identify and select information-rich cases in the most effective way in the face of limited resources, usually done through selecting individuals or groups of individuals knowledgeable about or experienced with a particular topic.^29,30^ Moreover, participant's availability, ability to communicate experiences and provide opinions in an articulate, expressive and reflective manner makes the purposive sampling strategy a good fit for qualitative research.^31,32^ We obtained a list of potential participants from the field offices of ADD International Bangladesh and their collaborators. Participants with lived experience of mental health problems, their family members and the community, as well as local government representatives, were purposefully selected for the study. These participants were selected to gain deep insights into the issues (e.g. risk factors and barriers) under study. We continued conducting interviews until no new information emerged.

### Units of study

The study utilized both interviews and focus groups to capture a comprehensive understanding of adolescent mental health issues by integrating personalized experiences with broader systemic perspectives. IDIs (n=12; mean age: urban 14.50 y, rural 13.33 y) provided an individualized setting for children and adolescents to share their personal experiences of mental health challenges. The WHO defines adolescence as the period of life between the ages of 10 and 19 y. This phase bridges childhood and adulthood and represents a critical stage of human development, essential for establishing the foundation for good health.^33,34^ Children and adolescents with diagnosed mental health conditions were identified and approached for an interview. The diagnoses were conducted by psychiatrists and we obtained the list of children and adolescents diagnosed with mental health conditions from the field offices of ADD International Bangladesh and their collaborators.

We conducted KIIs with caregivers (n=10; mean age 39.2 y), which offered critical insights into family dynamics, caregiving challenges and access to healthcare, allowing for a deeper exploration of their first-hand experiences. The two FGDs (n=16, with 8 participants in each group; mean age: urban 47.38 y, rural 41.5 y) facilitated interactive discussions among key stakeholders, including community representatives (i.e. local government officials), educators and healthcare providers, to identify systemic barriers and community-level challenges affecting mental health support. The selection of participants for either interviews or FGDs was guided by the nature of the information sought—IDIs and KIIs were used to gather detailed, personal narratives, while FGDs focused on broader policy, service accessibility and community perspectives. To ensure analytical rigor, data from interviews and FGDs were analysed separately before being triangulated to identify common themes and discrepancies, maintaining the integrity of individual and group insights. The codes that emerged from the data analysis included both personal lived experiences and collective viewpoints, providing a nuanced and well-rounded understanding of adolescent mental health challenges across different settings. The majority of the participants were of lower-middle socio-economic status and were Muslims. No participants approached refused to participate.

### Data collection method

Data were collected in Bangla, the state language of Bangladesh, by six community mental health workers (four males, two females). Each worker conducted one IDI and one KII. Additionally, four of the workers facilitated two FGDs, with two workers collaborating to conduct each FGD. The community mental health workers had training in basic counselling skills, however, they were trained about children’s and adolescents’ mental health problems, data collection procedures and cognitive interviewing prior to the commencement of data collection. Participants in the IDIs and KIIs were given the option to select the location of their interview, either at ADD International's office or their home, depending on their preference and convenience. FGDs were conducted in healthcare facilities. Interperson variabilities were addressed through training and standardization (role playing), pilot testing (conducting pilot interviews to identify variations in interviewing style) and regular meetings and debriefings to discuss experiences, challenges and potential discrepancies encountered during the interview. Interrater reliability was addressed by reviewing a subset of interviews by three interviewers to identify consistencies and differences in interpretation. Data were collected when the lockdown caused by the COVID-19 pandemic was lifted. However, necessary safety measures were followed during the data collection, such as using face masks and sanitizers and maintaining distance. In addition to the interviews and FGDs, we also incorporated observational data from community mental health workers and secondary data from ADD International's yearly records. Observational data included non-verbal cues such as body language and tone, which complemented the verbal responses in interviews and focus groups. Community mental health workers noted non-verbal cues that were recorded in field notes immediately after each session. ADD International's yearly records, which tracked mental health interventions and service utilization, were used to validate and contextualize the qualitative findings. These data sources were analysed separately and then triangulated to ensure consistency and provide a more comprehensive understanding of adolescent mental health issues.

### Data collection instruments and technologies

A topic guide was produced that included three major topic areas: mental health issues among children and adolescents, barriers to seeking mental healthcare and perception of mental health problems of children and adolescents living in rural and urban areas. The semi-structured interviews followed the topic guide, lasting for about 40 min. While the specific participants varied, the interview format remained semi-structured for all types of data collection methods, with open-ended questions designed to explore key themes. The questions were posed depending on the participant's responses.

Examples of the questions in the interview guide are, ‘What are the risk factors for mental health issues you have observed in children and adolescents aged between 5 and 18 years?’ and ‘What do you think are the barriers to seeking mental health care?’ Similar questions were asked during the FGDs. The interview guide was pretested on a sample of six people in the study areas (rural=3, urban=3) prior to the commencement of data collection to check the understanding of the questions, the order and the wording. The pretesting indicated that no changes were necessary for the questions in the interview guide. Interviews were collected between March and May 2021. Development of the topic guide, transcribing and analyses of the data were completed in the same period. Digital audio recorders were used to record the interviews.

### Data processing

The qualitative approach embraced in our research was thematic analysis.^35^ The research paradigm guiding our study was interpretivism.^36^ This paradigm is deemed appropriate because it focuses on understanding the subjective experiences and social contexts of participants. Interpretivism emphasizes the importance of context and meaning, which aligned with our study's aim to explore the sociocultural factors and perceptions influencing mental health issues and care access in Bangladesh. By utilizing thematic analysis within an interpretivist paradigm, our study aimed to provide a nuanced understanding of the complex sociocultural factors affecting mental health among children and adolescents in different settings in Bangladesh.

As previously noted, thematic analysis with an inductive approach was employed to analyse the data. This method was used to identify, analyse and report patterns (themes) within data, as it allowed for the extraction of key themes related to mental health issues, risk factors and barriers to accessing mental healthcare among children and adolescents. Usefulness and flexibility in allowing identification of recurring patterns or themes of meaning within the same dataset in relation to different epistemological and ontological positions are some of the key advantages of this approach.^35^ We manually analysed qualitative data. At the outset, the interview recordings were transcribed and the transcriptions were checked several times to ensure rigor and accuracy. We began by familiarizing ourselves with the data, which included reading through transcripts and notes multiple times to gain a comprehensive overview. During this process, we made initial notes and highlighted significant points. Next, we engaged in coding by identifying key phrases, sentences or sections and labelling them with descriptive codes.

Initially, data from interviews (both IDIs and KIIs) and FGDs were analysed separately, with thematic analysis identifying key themes for each type of data. To analyse the observational data, non-verbal cues such as body language and tone were coded for patterns that aligned with or contradicted verbal responses, providing deeper insights into participants’ emotional states or hesitations. ADD International's yearly records were reviewed for trends in mental health service utilization and intervention outcomes, allowing the qualitative data to be contextualized within broader programmatic trends. After the initial separate analyses, we triangulated the data by comparing insights from each data source to identify common themes and discrepancies. This process allowed us to integrate individual, family and community-level perspectives and validate the qualitative findings within broader programmatic trends, providing a more comprehensive understanding of adolescent mental health challenges.

We used Microsoft (Redmond, WA, USA) Word Document and Excel tools such as highlighters, coloured pens and sticky notes to differentiate themes and concepts. After coding, we organized similar codes into categories to form the coherent clusters. We developed themes by identifying patterns within these categories, organizing them into main themes and subthemes (Table 1).

**Table 1.** Organization of themes with broad and subcategories.

Themes	Category	Subcategory
Reported mental health issues	Internalizing mental health issues	Symptoms of anxiety
		Symptoms of depression
		Suicidal ideation
	Externalizing mental health issue(s)	Substance use
Key risk factors of mental health issues	Interpersonal risk factors	Family conflicts
	Social risk factors	Excessive use of mobile devices and the internet
		Easy availability of substances
Barriers to seeking mental health care	Knowledge and understanding	Low mental health literacy
	Access and resources	Limited access to mental health care
		Financial crisis
	Social and religious beliefs	Stigma and discrimination

### Techniques to enhance trustworthiness

Triangulation was used to indicate the varying experiences and perspectives of each group and dynamic contrast between data.^35^ Triangulation in our study involved collecting data through interviews, focus groups and observational notes and reviewing yearly records. The observational notes helped deepen the understanding of the participants’ emotional and behavioural states during the interviews and group discussions. ADD International's yearly records provided a historical context and allowed for comparison with the current findings, helping to corroborate the insights gained from the interviews and FGDs, enhancing the credibility and validity of the study's results.

We engaged multiple researchers to independently analyse the data and then compare their interpretations to further ensure the consistency and reliability of the conclusions reached. In addition, we also used member checking as an additional validation technique to ensure the accuracy and credibility of findings with the participants.^37^ After the initial data analysis, we presented the interpreted results to the study participants (n=5), asking them to review and confirm the accuracy of the findings. Our aims were to obtain feedback, clarifications and additional insights to ensure that their perspectives were appropriately represented.

### Researcher characteristics and reflexivity

Researchers involved in the study had training in qualitative research methodology and years of clinical experience in dealing with children and adolescent mental health issues. Researchers were actively engaged in the development of the interview guide and analyses of the data. In addition, researchers were aware of and sensitive to the cultural contexts in both rural and urban areas. Examples of cultural contexts included local customs, language and communication and social and religious beliefs as well as practices associated with mental health. Researchers were aware of the power dynamics to ensure comfortable sharing of information. Engaging local communities and stakeholders (e.g. collaborating with local healthcare workers, community leaders and local government representatives) was also adopted to gain trust and ensure the relevance and applicability of the research. In the present study, reflexivity was considered using notes taken during the interview (e.g. notes focused on participants’ comments and interviewers’ thoughts) and memos after the interview and during the data analysis process.

### Ethical considerations

The study was approved by the ethical review committee at the Department of Clinical Psychology, University of Dhaka (project ID: IR201101). An informed consent form explaining the nature and purpose of the study was given to all participants before the data collection. The potential risks and benefits of involvement, confidentiality, method of recording interviews and the right to withdraw at any time without consequences were also included in the consent form. Both written and verbal consent forms were obtained. Parental consent was also obtained for participants <18 y of age. A thumb mark was used to indicate consent for participants who were illiterate. Participation in the study was completely voluntary, therefore no reimbursement was provided. Data were stored while maintaining confidentiality. Researchers involved in the study had intensive training in the collection and storage of data. Data were recorded digitally and encrypted. Paper files were kept in secure storage. Identification numbers were used to label the data gathered from the participants and the remaining information deemed identifiable was stored separately from the data. We provided participants with a mental health service directory (https://add.org.uk/wp-content/uploads/2024/01/Bangladesh-Mental-Health-Service-Directory-in-English.pdf) to ensure participants could obtain mental health services during and after the interviews if needed.

## Results

The qualitative findings of the study produced three superordinate themes: mental health issues of children and adolescents, risks factors for children and adolescent mental health issues and barriers to seeking mental healthcare. Each superordinate theme contained both broad and subcategories.

### Reported mental health issues

This study showed the complex mental health landscape for children and adolescents in Bangladesh, uncovering both internalizing and externalizing mental health issues.

#### Internalizing mental health issues

Internalizing issues, such as symptoms of anxiety, depression and suicidal ideation, were prevalent, deeply impacting the psychological well-being of young individuals. Loneliness and loss of interest characterize symptoms of depression and suicidal ideation, while being anxious and sleeping less primarily characterize symptoms of anxiety.

A female adolescent said:

I feel alone and lost all the time. I have lost interest in what I used to like. Sometimes, I also feel empty and (I) think death is the best solution.

A female local representative observed:

Children and adolescents have become so anxious. They get anxious so easily. They avoid eating meals, sleep less, study less and sometimes stop talking to their parents.

#### Externalizing mental health issues

The internalizing mental health issues mentioned above were often compounded by externalizing issues (i.e. substance use), which not only posed direct risks but also exacerbated underlying mental health conditions. This highlights how these multifaceted problems are influenced by sociocultural and geographic dynamics.

A male adolescent said:

…to be honest with you I used to do drugs, for example, Yaba (methamphetamine) and cannabis…I still do, but occasionally at my friend's invitation. If I don't do drugs they will say I am not smart and have lost my manhood.

### Key risk factors for mental health issues

Several risk factors leading to mental health issues among children and adolescents were caused by interpersonal and social factors. Interpersonal risk factors include family conflicts, while excessive use of mobile devices and the internet and easy access to substances are social factors.

#### Interpersonal risk factors

Interpersonal risk factors (i.e. family conflicts) have contributed significantly to the internalizing mental health issues of anxiety, depression and suicidal ideation observed in youth.

An adolescent male reported:

…domestic violence and parental discord can make us feel really bad. If they see their parents quarrelling and fighting, they can also learn that. They may feel helpless, and this can affect their study and social relationship with others.

#### Social risk factors

Social factors, including the excessive use of mobile devices and the internet and the easy availability of substances, have further exacerbated these conditions and led to externalizing issues such as substance use disorders.

A male parent said:

…we allow children and adolescents to use mobile phones and access the internet. As we cannot always monitor and supervise what they are doing, this increases misuse and interferes with their study hours. They often make us buy internet packages. If you don't buy (internet packages), they will threaten you (for self-harm). Sometimes we (parents) don't know how to curtail the use and what to say.

A female healthcare provider said:

Young people have access to many drugs, for example, Yaba (methamphetamine) and cannabis. These (drugs) are cheap and can be found easily everywhere in the country, especially in the bordering areas (of India).

### Barriers to seeking mental healthcare

#### Knowledge and understanding

A key barrier to seeking mental healthcare was the lack of knowledge and understanding about mental health issues. Participants except for healthcare providers reported that they were not aware of the symptoms and severity of mental health conditions, leading to delayed recognition and treatment. In Bangladesh, this was compounded by low mental health literacy, where misconceptions and lack of awareness about mental health prevented people from acknowledging their need for help. This gap in knowledge means that symptoms of anxiety, depression and other mental health issues might often go unrecognized.

According to a male healthcare provider:

People do not have any understanding whatsoever about mental health, unlike physical health. The idea of mental health is absent in people. If any mental health conditions arise, they do not know who to reach out to and where they can find the treatment. Sometimes, people do not report symptoms properly and avoid them (symptoms) which we think are necessary to know about.

A female adolescent reported:

I do not know what that is because we never taught them in our school.

#### Access and resources

Limited access to mental healthcare resources was another critical barrier. In many regions, particularly rural areas, there is a scarcity of mental health professionals and facilities. This shortage makes it difficult for individuals to receive timely and adequate care. Additionally, economic constraints further limit access, as many families are unable to afford the costs associated with mental health services. In urban areas, where resources are available, the distribution is uneven, and participants do not have easy access to specialized care.

According to a male parent:

I am a poor man…I cannot afford to seek care in Dhaka or big cities. I am struggling with ensuring that my daughters receive education. If they have any physical health issue, Allah forbid if this a big one, I will find it difficult to get them treatment. For *mathar somossa* (problem in the head, an expression of how mental health is construed) I will not be willing to seek treatment.

A male community member in a rural area said:

We do not have mental healthcare facilities in our locality. So even if people know about mental health problems, they are not going to find it here. They have to go to Dhaka for that.

#### Social and religious beliefs

Social and religious beliefs also played a crucial role in deterring people from seeking mental healthcare. Mental health issues were stigmatized and often viewed through the lens of moral or religious failure. This stigma was reinforced by societal attitudes that labelled individuals with mental health conditions as weak or flawed and contagious. Religious beliefs such as mental health issues were a sin and caused by evil spirits, led people to seek help from faith-based healers instead of medical professionals, which delayed effective treatment. Individuals with mental health conditions were subjected to chaining, physical abuse and deprivation of their property. This stigma, discrimination and overreliance on non-medical interventions contributed to the perpetuation of mental health issues and hindered the development of a supportive environment for those in need.

A male community member said:

They took the child to a *kabiraj* (faith healer) for doing something religious (e.g., *jhar fuk* in Bangla) as they thought someone did a black magic. Sometimes they say it is a curse for their misdeeds.

A female local government representative said:

…it is so heart-touching that pregnant women are often discouraged to see the face of children with mental health needs or disabilities or their parents with a view to avoiding the same consequences.

A male community member said:

…children with mental illnesses are always deprived and underprivileged, neglected, they are considered as the burden of the family. They don't get to claim their rights or property.

## Discussion

This study aimed to offer the first qualitative exploration of mental health issues among children and adolescents in rural and urban Bangladesh. The findings revealed significant insights into reported mental health problems, risk factors and barriers to care. Our findings highlight both internalized and externalized mental health issues such as symptoms of depression, anxiety, substance use and suicidal ideation. These issues were closely linked to multiple risk factors, including problematic use of mobile phones and the internet, drug availability and family conflicts, with substantial barriers identified in accessing care, including stigma, mental health illiteracy and limited availability of services.

Our findings provide valuable insights into the lived experiences of children and adolescents with mental health conditions in Bangladesh and reflect themes that have been observed in existing literature on youth mental health globally.^38–40^ While our qualitative exploration does not allow for direct comparisons with prevalence estimates, the issues reported by participants regarding depression, anxiety and substance use align with broader patterns identified in previous research. For example, existing research indicates that around 3% of children have an anxiety disorder at any one time, which remains similar in adolescence, while the rates of depression are relatively low in children and increase to 3% in adolescence.^41^ The same study also reported that the cumulative prevalence of anxiety disorders and depression is 10% and 25%, respectively, by the age of 18 y. Although our sample was purposefully selected to include children and adolescents with diagnosed mental health conditions, the experiences they shared resonated with findings from cross-sectional studies in Bangladesh.^18,21–24,42–44^ For instance, >30% of adolescents in urban, semi-urban and rural areas have reported symptoms of depression^22,23^ while the prevalence of severe anxiety was about 20%.^24^ Spending substantial time on social media and residential settings (urban vs rural) were found to be risk factors for symptoms of both depression and anxiety. It is important to note that social media use, including problematic internet use, among adolescents has seen an upward trend over the years,^45–48^ a finding that is consistent with the symptoms identified in our study. Similarly, substance use was highlighted as a significant issue, aligning with estimates indicating that approximately 2.5 million people in Bangladesh, particularly those 15–30 y of age, experience drug addiction,^44^ and 1 in 10 adolescents report substance use disorders.^49^

Beyond the overlap in mental health issues, our study also underscores the interplay between these issues. Depression and anxiety often coexist and exacerbate each other, contributing to a complex cycle of mental health challenges.^50^ This interconnectedness is further complicated by the presence of substance use, which may serve as a maladaptive coping mechanism for adolescents dealing with depression, anxiety or suicidal thoughts. These findings resonate with the broader body of literature showing that mental health problems are rarely isolated and often co-occur, creating a web of distress that requires holistic intervention.^51^

The most striking element in our study was the significant role that social and cultural factors play in shaping mental health perceptions and behaviours. The stigmatization of mental health problems in both rural and urban areas emerged as a substantial barrier to care. The perception that mental health issues are caused by curses, sins or evil spirits, and the accompanying practices of physical abuse or deprivation of rights, reflect deeply rooted cultural beliefs that prevent timely intervention. These findings are consistent with previous studies indicating that stigma, particularly in LMICs such as Bangladesh, leads to delayed help-seeking and reliance on faith-based healing rather than professional care.^52,53^ Furthermore, this stigmatization often results in discriminatory behaviours, such as chaining or physical punishment, which compounds the isolation and suffering of affected individuals.^54^

The implications of these cultural factors are profound, as they not only delay access to effective treatment but also exacerbate mental health issues by reinforcing negative self-perceptions and contributing to social exclusion. This aligns with the existing literature, which emphasizes the need for culturally sensitive mental health interventions that address stigma and promote community-based mental health education.^55,56^

In addition to cultural barriers, financial constraints and limited access to services were also identified as significant barriers to seeking mental healthcare. Similar to findings in other studies,^53,57^ our participants noted that the high cost of mental healthcare and the lack of available services, particularly in rural areas, deterred them from seeking help. Furthermore, mental health literacy was identified as a critical issue, with many participants unable to recognize the symptoms of mental health disorders or understand the importance of professional help. These barriers, when combined, create a formidable challenge for accessing care, further compounding the mental health crisis among adolescents in Bangladesh.

Our study highlights the need for policy interventions that not only address the availability of mental health services but also tackle the cultural and economic barriers to care. Given the widespread stigma and lack of mental health literacy, interventions must involve community education, aimed at reducing stigma and improving understanding of mental health issues. Furthermore, mental health services need to be made more accessible, both in terms of affordability and availability, particularly in rural areas.

The mental health issues among children and adolescents in Bangladesh, particularly those related to depression, anxiety, substance use and suicidal ideation, are influenced by a complex interplay of individual, familial, societal and cultural factors. While our study provides rich qualitative insights into these issues, further research is needed to explore intervention strategies that are both culturally sensitive and accessible, ensuring that young people receive the care they need. The importance of early intervention, community involvement and addressing stigma cannot be overstated in improving the mental health outcomes of children and adolescents in Bangladesh.

### Study strengths and limitations

The study provides a comprehensive qualitative exploration of mental health issues among Bangladeshi youth from both rural and urban areas, offering rich, detailed insights into their experiences and perspectives in light of a wide range of experiences and contextual factors. This depth allows for a nuanced understanding of the specific challenges and barriers children and young people with mental health issues experience. The use of semi-structured interviews (KIIs and IDIs) and FGDs ensures a triangulation of data sources, which strengthens the validity and reliability of the findings. We utilized insights from various stakeholders such as community members, caregivers and local government representatives that provide a holistic view of the mental health landscape, highlighting systemic issues and potential areas for intervention. Finally, the study addresses the critical issue of stigma and discrimination associated with mental health, which is often underresearched in Bangladesh. This focus can inform culturally sensitive interventions aimed at reducing stigma irrespective of geographic location.

However, the study has a few limitations. For example, the sample size may be relatively small for capturing the full diversity of experiences and perspectives across Bangladesh. In addition, while the study covers both rural and urban areas, it is limited to specific regions (i.e. Bagerhat, Dhaka and Jashore), thus the findings may not be representative of other regions in Bangladesh with different sociocultural dynamics. The qualitative approach, while rich in detail, may not provide the statistical generalizability that quantitative studies offer. The findings are context-specific and may not be applicable to all populations or settings across Bangladesh. The subjective nature of qualitative research means that findings can be influenced by the researchers’ perspectives and interpretations. Efforts to mitigate this, such as member checking and triangulation, were employed, but they may not eliminate all biases. Furthermore, the reliance on self-reported data can introduce bias, as participants might underreport or overreport their experiences due to social desirability or recall bias.

### Clinical and research implications

The findings can inform policymakers about the specific mental health needs of Bangladeshi youth, leading to the development of targeted mental health policies and programs that address both urban and rural contexts. Additionally, insights into the barriers to mental healthcare, including stigma and sociocultural beliefs, can guide the design of culturally sensitive interventions that are more likely to be accepted and effective in the Bangladeshi context. The study highlights the need for educational campaigns to improve mental health literacy and reduce stigma. Such campaigns could be tailored to different community settings, leveraging local beliefs and practices to promote mental health awareness. Identifying the barriers to mental health service provision can help in strategizing ways to improve access, such as training more mental health professionals, setting up more mental healthcare facilities and integrating mental health services into primary healthcare. Finally, the study lays the groundwork for future research by identifying key areas of concern and highlighting the importance of qualitative exploration in understanding mental health issues. Subsequent studies could build on these findings with larger, more diverse samples and mixed method approaches to validate and extend the insights.

## Conclusions

The study suggests that mental health issues among children and adolescents in Bangladesh are significantly influenced by a combination of sociocultural factors, including pervasive stigma and discrimination. The qualitative exploration revealed symptoms of anxiety, depression, substance use and suicidal ideation as prevalent mental health concerns. Barriers to seeking mental healthcare, such as low mental health literacy, limited access to services and detrimental social and religious beliefs, further exacerbate these issues. The findings underscore the necessity for culturally sensitive interventions and improved mental health education to address these barriers. By highlighting these challenges and needs, the study provides a foundation for developing targeted mental health policies and programs tailored to the unique contexts of rural and urban Bangladesh.

## Data Availability

Data and codes will be provided upon request.
